# A13 MICROBIOTA FROM PATIENTS WITH INFLAMMATORY BOWEL DISEASE (IBD) INDUCE ALTERED BEHAVIOUR AND VISCERAL HYPERSENSITIVITY IN MICROBIOTA-HUMANIZED MICE

**DOI:** 10.1093/jcag/gwad061.013

**Published:** 2024-02-14

**Authors:** F A Vicentini, J Pujo, G H Rueda, A Nardelli, G De Palma, E Verdu, S Collins, P Bercik

**Affiliations:** McMaster University, Hamilton, ON, Canada; McMaster University, Hamilton, ON, Canada; McMaster University, Hamilton, ON, Canada; McMaster University, Hamilton, ON, Canada; McMaster University, Hamilton, ON, Canada; McMaster University, Hamilton, ON, Canada; McMaster University, Hamilton, ON, Canada; McMaster University, Hamilton, ON, Canada

## Abstract

**Background:**

IBD is characterized by relapsing episodes of tissue-damaging inflammation in the gastrointestinal tract. Abdominal pain is common in IBD and often persists in the absence of overt inflammation. The treatment has limited efficacy, in part due to our partial understanding of its pathophysiology, and thus commonly leads to dependency on opioids. Moreover, patients with IBD are more likely to develop psychiatric comorbidities (anxiety and depression), further impairing the disease course and quality of life. The gut microbiota has been shown to modulate pain, mood, and anxiety. Gut bacterial composition and function are altered in patients with IBD. However, whether IBD microbiota affects pain perception and emotional behaviour is unknown.

**Aims:**

To determine whether microbiota from patients with IBD contributes to visceral hypersensitivity and behavioural abnormalities in a microbiota-humanized mouse model.

**Methods:**

Adult germ-free C57BL/6 mice (n=65) of both sexes were colonized with fecal microbiota from 7 patients with IBD (Crohn’s Disease n=5, Ulcerative Colitis n=2) and 3 healthy controls (HC). Mice were fed with a human-like (Western) diet. Behavioural tests (light preference, open field, and tail suspension tests) were employed to assess anxiety- and depression-like behaviour. Visceral sensitivity was assessed by measuring visceromotor responses to colorectal distension.

**Results:**

Compared to mice with HC microbiota, mice with IBD microbiota had reduced body weight gain. However, no differences in colon length were found, suggesting that IBD microbiota did not induce overt colonic inflammation. Mice with IBD microbiota displayed a reduced preference for the light chamber and increased immobility in the tail suspension test compared to HC mice, suggestive of anxiety- and depression-like behaviour, respectively. Importantly, no differences in overall locomotion were observed, thus ruling out sickness-like behaviour. When subjected to colorectal distension, mice with IBD microbiota had an overall higher visceromotor response (area under the curve) compared to HC mice. Furthermore, they displayed increased responses to non-noxious stimuli (100 μL distension), suggestive of visceral allodynia.

**Conclusions:**

Our data demonstrate that microbiota from patients with IBD negatively affects the gut-brain axis, by inducing changes in emotionality-related behaviour and triggering visceral hypersensitivity. Uncovering the microbial products and molecular mechanisms underlying these abnormalities will guide the development of novel microbiota-based therapies for patients with IBD.

**Funding Agencies:**

CCC, CIHRCrohn's and Colitis Foundation (USA)

